# Direct observational evidence of an oceanic dual kinetic energy cascade and its seasonality

**DOI:** 10.1126/sciadv.abq2566

**Published:** 2022-10-12

**Authors:** Dhruv Balwada, Jin-Han Xie, Raffaele Marino, Fabio Feraco

**Affiliations:** ^1^ Lamont-Doherty Earth Observatory, Columbia University, New York, NY, USA.; ^2^ Department of Mechanics and Engineering Science at College of Engineering and LTCS, Peking University, Beijing 100871, P. R. China.; ^3^ Joint Laboratory of Marine Hydrodynamics and Ocean Engineering, Pilot National Laboratory for Marine Science and Technology (Qingdao), Shandong 266237, P. R. China.; ^4^ Univ Lyon, CNRS, École Centrale de Lyon, INSA Lyon, Univ Claude Bernard Lyon 1, Laboratoire de Mécanique des Fluides et d’Acoustique, UMR5509, F-69134 Écully, France.

## Abstract

The ocean’s turbulent energy cycle has a paradox; large-scale eddies under the control of Earth’s rotation transfer kinetic energy (KE) to larger scales via an inverse cascade, while a transfer to smaller scales is needed for dissipation. It has been hypothesized, using simulations, that fronts, waves, and other turbulent structures can produce a forward cascade of KE toward dissipation scales. However, this forward cascade and its coexistence with the inverse cascade have never been observed. Here, we present the first evidence of a dual KE cascade in the ocean by analyzing in situ velocity measurements from surface drifters. Our results show that KE is injected at two dominant scales and transferred to both large and small scales, with the downscale flux dominating at scales smaller than ∼1 to 10 km. The cascade rates are modulated seasonally, with stronger KE injection and downscale transfer during winter.

## Teaser

Ocean observations show wave and turbulence merge and split flows, simultaneously transferring energy to large and small scales.

## INTRODUCTION

The oceanic circulation is primarily forced at scales of O(1000) km by the winds, tides, and solar heating and is dissipated by friction at scales of O(1) mm. Ocean turbulence helps to redistribute the energy across scales, populating the range between forcing and dissipation scales and also promoting the exchanges between potential and kinetic energy (KE) reservoirs (*1*). The bulk of the oceanic KE resides in flows with horizontal scales of hundreds of kilometers (*2*), the so-called mesoscales. These flows are characterized by rapid rotation and strong stratification, so their dynamics are well described by the quasi-geostrophic (QG) theory. These dynamics dictate a general tendency of the oceanic flow to transfer KE from small to large scales through the inverse cascade (*3*, *4*) and suggest a rather steep drop-off in KE at smaller scales, following a *k*^−3^ spectrum. Consequently, according to QG dynamics, the mesoscale KE is expected to be dissipated primarily by boundary friction. However, estimates suggest that dissipation through boundary friction accounts for only 1/10th of the total KE injection, raising a puzzle about the dissipation mechanisms (*5*). To resolve this puzzle, there must be mechanisms not described in the QG theory for transferring KE from large to small scales, through a forward cascade.

Observations and high-resolution simulations have shown that the range of scales referred to as the submesoscales, scales between three-dimensional (3D) turbulence [O(50 to 100) m] and the mesoscale [O(100) km], are quite energetic in the surface ocean (*6*, *7*), often following a *k*^−2^ spectral slope for horizontal KE and buoyancy variance, and show a pronounced seasonal modulation in KE levels (*8*–*11*). These scales are thought to be energized through mesoscale-driven straining of buoyancy fronts (*12*, *13*) or mixed-layer instabilities (*14*, *15*), which act to release the available potential energy stored in mixed layers. The latter mechanism is now routinely implicated for the observed seasonality at these scales. The shallow spectral slope implies that the Rossby number, a ratio of inertial force to the Coriolis force, can become O(1) at these scales, which suggests that the submesoscale flows can escape the constraints of geostrophic balance and potentially transfer KE to smaller scales (*16*, *17*). These flows are also expected to play an important role in the mixed-layer restratification (*18*) and transporting tracers between the mixed layer and the interior (*19*, *20*).

The suggestion that submesoscale flows at the surface can result in a forward cascade of KE has been confirmed in high-resolution ocean models (*21*, *22*). These simulations show that the surface KE flux can undergo a dual cascade, flowing upscale at large scales and downscale at small scales, with the forward cascade being present at scales roughly smaller than O(10) km. The KE reservoir is supplied by conversion from available potential energy to KE, and the ageostrophic flow is crucial for the forward cascade to emerge. In this phenomenology, the surface KE does not develop an inertial range, as it is not a conservative quantity because of exchanges with the underlying interior (*16*). In addition, direct numerical simulations of rotating stratified turbulent flows in the appropriate parameter regimes (*23*–*25*) have indicated that a dual KE cascade can also be sustained in the ocean interior. While evidence and mechanistic understanding of the dual cascade in the ocean has been made possible by sophisticated high-resolution simulations, there are no observational studies that have been able to unambiguously confirm its presence yet.

Estimating the interscale energy transfers in the ocean from observations is extremely challenging because of the fact that conventional spectral flux estimation methods used to analyze numerical simulations (*26*) require the availability of synoptic measurements on a regularly sampled grid and over a fairly large region; technology to collect these measurements at submesoscales is not available at this time (*17*). Sea surface height (SSH) measurements from satellites come the closest to producing datasets that are amenable to spectral flux calculations; they provide gridded estimates of surface geostrophic velocity with a nominal spatial resolution of O(100) km and time resolution of a week. Spectral flux calculations from SSH-based velocities have provided clear evidence for the presence of an inverse KE cascade at scales larger than 100 km and suggested the presence of a forward KE and enstrophy cascade at scales smaller than 100 km (*27*, *28*). However, the conclusions about scales smaller than 100 km are debatable because these estimates rely on the assumption of geostrophy to estimate the velocity from SSH and because of strong sensitivity to the gridding and interpolation methods (*29*). In coastal locations, high-frequency radars have been used to measure the surface velocity fields with resolutions of a few kilometers and can be used for estimating the spectral flux, but they are limited in coverage to within ∼10 to 100 km off the coast (*30*). Velocity estimates from a mooring array were used in a recent study to suggest the presence of a forward cascade at the submesoscales in the spring (*31*) using frequency decomposition. However, this work relied on assuming a simple relationship between the temporal and spatial scales and did not directly probe the structure of how KE is transferred across spatial scales.

An approach to investigate the properties of the interscale KE transfers from observations consists of using third-order velocity structure functions (SF3; see Methods), which can be estimated from ungridded or scattered measurements under the assumption of statistical homogeneity. Surface drifters, released in large clusters, have allowed for this analysis of the KE cascades (*32*–*34*). These studies, all from drifter releases in the northern Gulf of Mexico (GoM), have suggested that a forward cascade exists at scales smaller than O(1 to 10) km based on a sign reversal in SF3 around these scales and have quantified the forward KE flux based on exact formulae for the SF3 derived using inertial-range arguments (*26*). A major improvement of this methodology for the quantification of the KE fluxes is due to Xie and Bühler (*35*), who proposed a forcing scale–resolving SF3 formulation able to capture the simultaneous bidirectional KE transfer. This formulation applies to a range beyond the inertial range, and its implementation does not require the identification of inertial ranges to fit SF3 expressions with power functions based on inertial range arguments. Meanwhile, it also allows the estimation of the KE injection, which is important for gaining a better understanding of the turbulent cascades.

Here, we apply these new theoretical insights to two surface drifter datasets, collected during the Grand LAgrangian Deployment (GLAD) and the LAgrangian Submesoscale ExpeRiment (LASER) in the GoM in summer and winter, respectively. We focus specifically on velocity estimated from surface drifter trajectories in the northern GoM, in waters that are deeper than 500 m and away from the continental shelf. Our results confirm the presence of a seasonal modulation of submesoscale surface KE and characterize and quantify the interscale KE transfer simultaneously to the large and small scales, at the submesoscales in the ocean. The results confirm the existence of a dual cascade of KE at the ocean surface, which is found to be energized primarily at scales close to the mixed-layer and interior deformation radii, with the change in the direction of KE flux happening near a scale where the local Rossby number is O(1). The KE injection and flux are stronger in winter than in summer, and the scale of KE injection that likely corresponds to the mixed-layer instability shifts to larger scales in winter relative to summer.

## RESULTS

### GLAD and LASER experiments

**Fig. 1.**
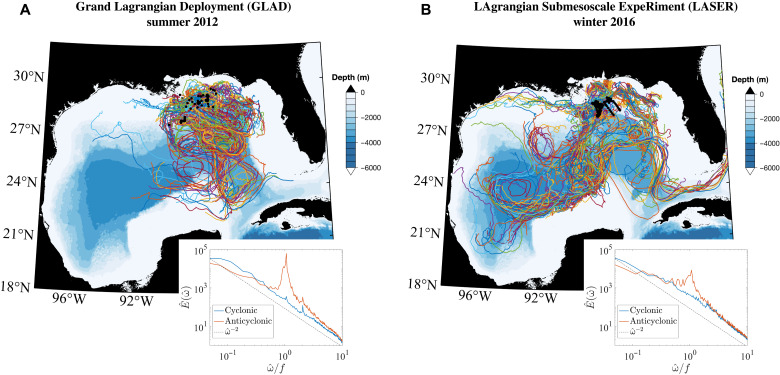
Spatial distribution of the drifters. Drifter tracks and the Lagrangian frequency spectra (inset plots) for the GLAD (**A**) and LASER (**B**) experiments. Black dots indicate the deployment locations, and the colored contours indicate the bathymetric depth. In our study, we only considered the sections of the drifter tracks that were in water deeper than 500 m and in between east of 84°W to 91°W and north of 24°N. The inset plots show the rotary frequency power spectrum plotted as a function of frequency ($\hat{\omega }$) normalized by the Coriolis frequency (*f*), where the power spectra were estimated as an average over spectral estimates from 28-day long segments of the drifter trajectories.

We analyze data from surface drifters that were deployed in the northern GoM, in a region usually to the north of the Loop Current. These surface drifters tracked the flow in the top 0.6 m of the water column (*36*) and were deployed as part of the GLAD experiment in Summer/July to August 2012 (*37*) and the LASER experiment in Winter/January to February 2016 (*38*), which are, to date, the largest simultaneous drifter deployments. Over the course of the 3 to 4 months that the drifters were active, they dispersed to span a large part of the GoM, and this long-term dispersion was largely influenced by the basin-scale and mesoscale circulation in the region (Fig. 1). The part of the data analyzed here comes mainly from the initial few weeks after the deployments when the drifters are relatively close to each other and are present mostly in the northern GoM. In addition, we excluded the drifter tracks that were in waters shallower than 500 m or ventured west of 91°W, east of 84°W, or south of 24°N, as we want to focus on the dynamics away from the continental shelf and in the northeastern GOM.

The atmospheric forcing during the summer deployment was characterized by relatively weak winds (∼5 m/s), while the winter deployment experienced stronger winds (∼8 m/s) and severe storms. The summer months in the northern GOM are also characterized by very shallow mixed layers (∼10 m) and lateral buoyancy gradients that are produced by the inflow of fresh water from the Mississippi River delta, while in winter, the mixed layer deepens (∼80 m) and the lateral buoyancy gradients are primarily a result of temperature variations (*34*, *39*). The summer drifter trajectories and velocities show a marked presence of inertial oscillations (Fig. 1, inset), and the amplitude of these oscillations is damped by about an order of magnitude in winter, likely in response to the seasonal modulation of the mixed-layer depth (*40*). In this study, the length scale–wise variation of the KE content in the surface ocean is assessed by means of the SF2, and a novel approach based on the SF3 is applied to get insights into the transfer and injection of KE as a function of length scales.

### Seasonal modulation of surface KE

The SF2 ($D_{LL}(r)=\langle \delta u_{L}^{2}\rangle ,D_{TT}(r)=\langle \delta u_{T}^{2}\rangle$; see Methods for details) reflects how KE is distributed as a function of scale; SF2 behaves roughly as a cumulative sum of KE up to a particular scale, and larger SF2 values suggest greater levels of KE at scales near and smaller than a particular scale. The detailed definition of SF2 and its relationship to the KE power spectra can be found in Methods.

**Fig. 2.**
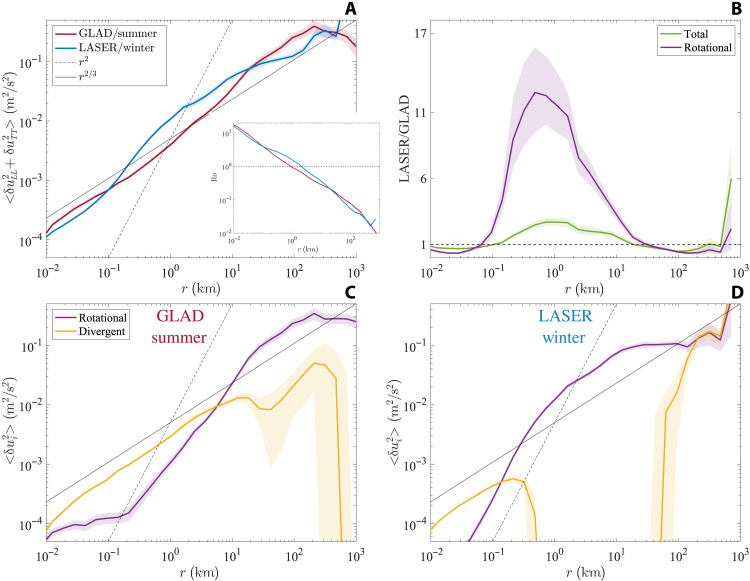
Distribution of KE as a function of scale represented using SF2. (**A**) The total, sum of the longitudinal and transverse, SF2 (*D*_tot_(*r*) = *D_LL_*(*r*) + *D_TT_*(*r*)) as a function of separation scale for the GLAD and LASER experiments. The inset shows the Rossby number, defined as $\text{Ro}(r)=\sqrt{D_{\text{tot}}(r)}/fr$, as a function of scale, with the horizontal line indicating Ro = 1. (**B**) The ratio of total and rotational SF2 from the LASER and GLAD experiments, with dashed horizontal line indicating a ratio of 1. (**C** and **D**) The decomposition of the total structure function into the rotational and divergent components, into Helmholtz decomposition, and for the GLAD and LASER experiments. Thin gray lines in (A), (C), and (D) indicate power laws with exponents of 2 and 2/3, which would theoretically correspond to an enstrophy cascade and an energy cascade, respectively.

The surface drifter SF2 shows a marked change in properties between summer and winter (Fig. 2). The total SF2 (*D*_tot_ = *D_LL_* + *D_TT_*), reflective of the total horizontal KE, in winter is larger by a factor of approximately 2 at scales on the O(100 m to 10 km), while the nondivergent or rotational part of SF2 (discussed more in the next paragraph) in winter is around 10 times larger at those scales (Fig. 2B) compared to summer. Larger scales, O(20 to 100 km), show a slight reduction of total SF2 in winter; this is most likely a reflection of the synoptic modulation of the mesoscale eddies resulting from their chaotic variability rather than the seasonal variability. The smallest scales, O(<100 m), also show a slight reduction of total SF2 in winter, which is likely related to precise deployment conditions and spatial variability because these smallest range of scales are sampled for very short periods after deployment. We also defined a scale-dependent Rossby number using the total SF2, as $\text{Ro}(r)=\sqrt{D_{\text{tot}}(r)}/fr$, with *f* being the Coriolis frequency and *r* being the separation scale. This Rossby number is O(1) at scales smaller than 1 to 5 km and is slightly greater in winter than summer following the seasonal modulation of the total SF2.

To gain further insight into the type of the flows that contribute to the total SF2, we decomposed it into the rotational (*D*_R_) and divergent (*D*_D_) contributions using a Helmholtz decomposition (*41*). The relative contribution from the divergent and rotational parts of the flow changes notably between seasons and indicates a marked variation in the flow dynamics between summer and winter (Fig. 2, C and D). In summer, the divergent motions dominate up to scales of 5 km, while in winter, the dominance of divergent motions is limited to scales smaller than about 100 m. The power law behavior of the rotational SF2 also shows a marked change between seasons. While both seasons show that the rotational SF2 has a shallow slope at large scales, suggestive of an inverse energy cascade, and has a steeper slope at smaller scales, the scale where this slope changes shifts from approximately 1 km in winter to around 20 km in summer.

The SF2 indicates that the submesoscale flow, roughly defined as scales smaller than 50 km, is more energetic in winter than in summer, and this is primarily a result of strengthening of the rotational (nondivergent) flow as the divergent flow weakens. The change in power law behavior of the rotational SF2 between seasons suggests that a higher amount of KE is injected into the nondivergent part of the flow near the mixed-layer deformation radius, O(1 to 10 km), in winter, and the cascade of this energy is strong enough to paint the distribution of KE in the submesoscales. It is also notable that the energized winter flow is primarily rotational, which suggests a dominance of geostrophically balanced flows likely energized by mixed-layer instability, while the summer flow has a large contribution from divergent component, suggesting an abundance of ageostrophic motions, potentially resulting from strain-driven frontogenesis and internal waves (also highlighted in the Lagrangian frequency spectra in Fig. 1). These results from the surface drifters suggest that the seasonality of the surface flows in the northeastern GoM is qualitatively similar to other parts of the ocean that have a large seasonal modulation of mixed-layer depth (*7*, *15*).

### Seasonality of interscale KE transfers

**Fig. 3.**
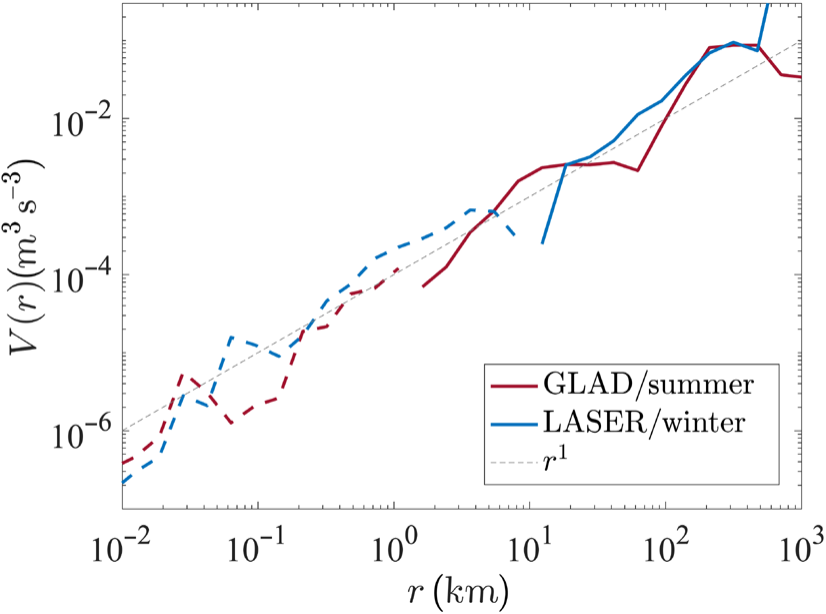
SF3 ($V(r)=\langle \delta u_{L}^{3}\rangle +\langle \delta u_{L}\delta u_{T}^{2}\rangle$) from GLAD and LASER. The absolute value of *V*(*r*) is plotted, and the range of scales where *V*(*r*) is negative is indicated as dashed lines. A linear power law is also indicated as a dashed line for reference. Error estimates are not plotted here because they cannot be represented properly on the logarithmic axis near the scale where the sign changes; the error estimates on these quantities can be seen in Fig. 4 (A and D).

The SF3 ($V(r)=\langle \delta u_{L}^{3}\rangle +\langle \delta u_{L}\delta u_{T}^{2}\rangle$) is a metric that can be roughly associated with the turbulent KE transfer rate (*26*), and their sign, under a certain hypothesis, is associated with the direction of the KE transfer, with a negative SF3 indicating a forward (or downscale) transfer of energy and a positive SF3 indicating an upscale energy transfer. The GLAD experiment, conducted in summer, provided the first observational evidence in the ocean that a wide range of scales, O(<1 km), had a negative SF3 (*32*, *33*, *42*), which is emblematic of a forward cascade of KE. The LASER experiment conducted a few years later in the winter season further solidified the generality of this observational result (*34*, *43*) and also showed a seasonal modulation in the length scale where the sign change happens (Fig. 3). A similar result showing negative values of SF3 at smaller scales had been observed in the atmosphere about two decades earlier (*44*) and was recently observed in the eastern Pacific Ocean (*45*).

**Fig. 4.**
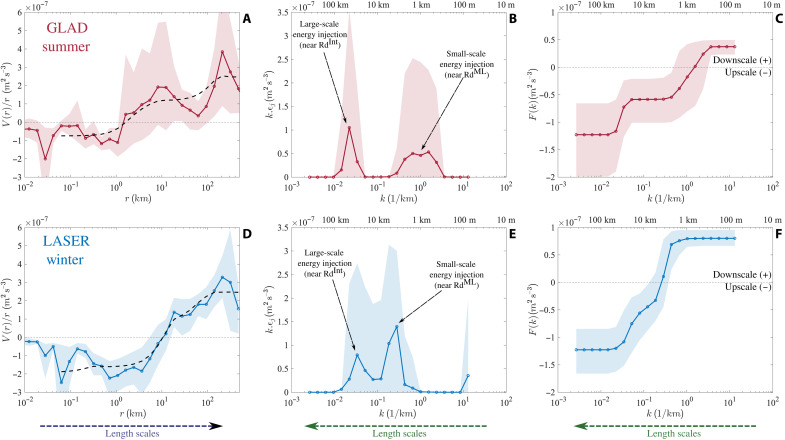
Estimates of spectral flux and corresponding parameters from SF3 for the GLAD/summer and LASER/winter experiments. The top and bottom panels are obtained from GLAD and LASER, respectively. (**A** and **D**) The normalized SF3 [*V*(*r*)/*r*] and the fit, dashed black line, over scales of 50 m to 500 km. (**B** and **E**) The estimated energy injection rate (ϵ*_j_*) as a function of wave number, plotted in a variance-preserving form to account for the logarithmic *x* axis and to ensure that these panels can be visually interpreted such that the amount of energy injected corresponds to the area under the curve. (**C** and **F**) Spectral flux [*F*(*k*)] as a function of wave number. The positive and negative values in (C) and (F), as highlighted by text markers, denote downscale and upscale fluxes, respectively. Note that the length scales increase from left to right in (A) and (D), where the *x* axis corresponds to separation scale, while the length scales increase from right to left in the other panels, where the *x* axis corresponds to wave number; this is represented by arrows near the bottom of the figure. The scales with large amount of energy injection referred to as large-scale and small-scale peaks in the text are marked in (B) and (E) and as discussed in the text are likely associated with the interior deformation radius (Rd^Int^) and mixed-layer deformation radius (Rd^ML^), respectively. At the top of (B), (C), (E), and (F), we have denoted the length scales corresponding to the wave numbers on the *x* axis, where the length scale is defined as 1/*k*.

The interpretation, in these previous studies, of the direction of KE transfer based on the sign of the SF3 is rooted in classic inertial-range theories, e.g., Kolmogrov’s 4/5 law (*46*). These interpretations are potentially suspect, or are at least only qualitatively correct, when inertial ranges cannot be clearly identified or when the assumptions used to reach inertial range arguments, e.g., purely 2D or 3D flow or asymptotic separation from forcing scales, are not satisfied. To overcome this major limitation, we use a new theoretical framework developed by Xie and Bühler (*35*) that allows us to directly infer the spectral fluxes from SF3, under the conventional assumptions of isotropy and homogeneity. In this framework, the spectral flux [*F*(*k*)] is expressed by the corresponding KE injection rates [ϵ(*k*)] at wave number *k* (corresponding to scale *l* = 1/*k*) and the upscale KE transfer rate (ϵ*_u_*). This spectral flux is analytically transformed to the corresponding SF3 in terms of the same parameters. The observational estimate of the SF3 can be fit using this analytical form, and all the parameters and thus the corresponding spectral flux can be inferred. To avoid amplifying small-scale error in the inversion process, we made a physically motivated and pragmatic assumption that, over the range of observed scales, the KE injection is positive and only adds KE to the surface flow, which is equivalent to assuming that the spectral flux is an increasing function of wave number and that all the dissipation and extraction of KE out of the surface flow takes place outside the range of scales where the fitting is done. This is very well justified for dissipative mechanisms, which are active at scales much smaller than the ones observed here, but any transfer from KE to potential energy over the range of fitting scales has been ignored here (further details in Methods and the Supplementary Materials). Even with this assumption, the fitted SF3 matches the observed SF3 relatively well, capturing the broad structure within error bars and without fitting every small detail (Fig. 4, A and D).

The estimated parameters provide us with two crucial physically relevant measures of the flow dynamics, the distribution of KE injection rate (Fig. 4, B and E) and the spectral flux as a function of length scale or wave number (Fig. 4, C and F). The KE injection shows two distinct peaks, one associated with smaller scales and another with larger scales. The large-scale peak occurs around 40 to 50 km, spreading between 20 and 100 km, and does not vary significantly with the season. In contrast, the smaller-scale KE injection is modulated seasonally. In summer, this KE injection peaks around 1 km and is spread between 500 m and 5 km, while in winter, this peak is shifted to about 5 km and is spread between 1 and 10 km. In summer, the small- and large-scale peaks are distinct, while in winter, the small-scale KE injection is stronger in amplitude than summer and the small- and large-scale KE injections show a tendency to partially overlap. The scale where the spectral flux changes from being negative (upscale transfer) to positive (downscale transfer) is also approximately the same as the scale where the small-scale KE injection peaks, which is further correlated with the scale where the Rossby number starts to become O(1) (fig. S2A, inset).

Quantitative estimates of the upscale KE transfer rate and KE injection rates are summarized in Table 1. The upscale KE transfer rate, the most negative value of the spectral flux (Fig. 4, C and F), and the KE injection at larger scales are similar within error bars between the two seasons, while the KE injected at smaller scales is enhanced by about 25 to 30% in winter. In summer, about 75% of the total injected KE, summed over both the large- and small-scale injections, is transferred upscale, while in winter, this ratio is reduced to about 60%, indicating a strengthening of the forward cascade. Note that in both summer and winter, a fraction of the KE injected at the smaller KE injection scale is transferred upscale. Considering the almost unchanged upscale KE transfer rate (reflective of KE flux to scales greater than 100 km), most of the extra KE injected in winter relative to summer transfers downscale.

**Table 1.** Estimated upscale KE transfer rate (ϵ*_u_*), large-scale KE injection rate $\left(\sum_{k_{j}=1/100\text{km}}^{k_{j}=1/10\text{km}}\epsilon_{j}dk\right)$, and small-scale KE injection rate $\left(\sum_{k_{j}=1/10\text{km}}^{k_{j}=1/100m}\epsilon_{j}dk\right)$. The values shown are the mean, and the range in the parenthesis are the 5th and 95th percentiles.

**Experiment/> season**	**Upscale KE transfer rate, ϵ_*u*_ (m^2^/s^3^)**	**Large-scale KE injection rate (10 to 100 km) (m^2^/s^3^)**	**Small-scale KE injection rate (100 m to 10 km) (m^2^/s^3^)**
LASER/winter	1.23 (0.85 – 1.66) × 10^−7^	0.78 (0 – 1.36) × 10^−7^	1.25 (0.84 – 1.73) × 10^−7^
GLAD/summer	1.23 (0.66 – 1.99) × 10^−7^	0.64 (0 – 1.56) × 10^−7^	0.96 (0.53 – 1.41) × 10^−7^

The estimation of interscale KE transfers from the observed SF3 indicates that the range of scales from 100 m to 500 km are energized primarily at two distinct scales, which are roughly analogous to the interior deformation radius, O(50 km), and the mixed-layer deformation radius, O(1 to 10 km). The KE injection near the mixed-layer deformation radius is seasonally modulated, with the injection rate and scale increasing slightly in winter. This is most likely due to a deepening of the mixed layer, which is, in turn, associated with more available potential energy for release and a larger scale of instability. About 25 to 40% of the KE injected into the system undergoes a forward cascade at scales where the Ro > O(1), likely because the flow starts to escape the strong constraint imposed by rotation for KE to be transferred upscale. The flow in winter has a higher propensity to cascade KE to smaller scales, which may be understood as the more energetic flow in winter having higher speeds and thus a greater likelihood to escape rotational effects. These results provide the first direct estimate of the downscale KE transfer rate, O(10^−7^m^2^/s^3^), at the submesoscales. This estimate for the KE transfer rate is similar to those retrieved for the KE dissipation in the mixed layer using microstructure estimates (*47*), suggesting that the submesoscale flows can provide a direct pathway toward dissipation.

## DISCUSSION

The observations of submesoscale turbulence at the surface ocean, in the northern GoM, show the presence of a dual KE cascade; KE dominantly cascades toward small scales at scales smaller than O(1 to 10 km) and toward large scales at scales larger than O(1 to 10 km). The KE injection takes place primarily over two distinct scale ranges, at smaller scales O(500 m to 10 km) near the mixed-layer deformation radius and at larger scales O(20 to 50 km) near the interior deformation radius. The presence of a turbulent dual KE cascade has been hypothesized in the literature as a mechanism needed to accomplish the small-scale dissipation in the ocean. This hypothesis has stemmed from high-resolution ocean models and direct numerical simulations of rotating stratified flows, but this is the first time it has been directly confirmed in ocean observations using the estimates of SF3 analyzed with an innovative methodology.

The strength of the KE transfers and the scales at which the dual cascade develops are modulated seasonally, likely in response to the change in mixed-layer depth and strength of lateral buoyancy gradients set by the atmospheric forcing and freshwater river outflow. The net KE injection over the observed range of scales and the fraction of this KE cascading forward to smaller scales are enhanced in winter, when the mixed layer is deeper. Although the KE injected at small scales increases in winter, this extra KE injection mainly leads to a strengthening of the downscale flux, without affecting the upscale KE flux at scales larger than 50 km. The scale at which the net KE flux switches from being dominantly toward small scale to dominantly toward large scale seems to be correlated with the scale at which the local (in scale) Rossby number becomes O(1), the latter shifting toward larger scales in winter as the net KE in the system increases.

A seasonal modulation in the quasi-equilibrium distribution of KE over scales is also observed, as evidenced by the SF2 analysis and its Helmholtz decomposition. In winter, there is more KE relative to summer in the flow over the submesoscale range, and also this range of scales is dominantly composed of nondivergent motions. This suggests that the enhancement and changes in flow structures in winter could likely be tied to a mechanism such as mixed-layer instability, which strengthens in the presence of deeper mixed layers and would largely energize the geostrophically balanced part of the flow. In summer, divergent motions account for a large part of the KE over the submesoscale range, suggesting that the internal waves enhanced in amplitude by the shallower mixed layer or strain-driven ageostrophic frontogenesis are dominant.

Our analysis focused on observations from the northern GoM, particularly the deeper ocean away from the continental shelf where the seasonal modulation in mixed-layer depth is quite large. However, we believe that the presence of a dual KE cascade is likely to be a ubiquitous feature of submesoscale turbulence in the global ocean, and its particular properties, such as the scale at which the net flux changes sign, would be modulated by the local environment. Our results would indicate that if the Ro > O(1) at any scales in a region, then a forward cascade would likely ensue, which is possible as the dynamics would diverge from the traditional QG phenomenology. This is likely to be the case for most of the surface ocean according to high-resolution simulations (*48*), which suggest that the submesoscale range of scales are more energetic than what QG dynamics would suggest in most places, and the particular dynamical contributions to these scales, geostrophically balanced motions versus internal gravity waves, are modulated seasonally and spatially. It is worth noting that these high-resolution simulations are far from being converged, and the departure from QG theory is likely to be even more stark as finer scales are resolved and in the real ocean.

This discovery of a well-defined dual KE cascade presented here rests on two key elements: high-density sampling of the surface flows using a dense array of surface drifters (*36*, *37*) and theoretical advancement in structure-function analysis by generalizing the use of SF3 (*35*). However, as most observational analyses go, our results require assumptions and are likely to have some biases that are important to discuss, as they will chart the path for future research. The theory surrounding structure functions is developed in Eulerian coordinates and assume homogeneity and isotropy, while the surface drifters sample the velocity field following the horizontal flow, with the real ocean having some degree of spatial inhomogeneity and anisotropy introduced by the complexity of the domain or forcing mechanisms. It has been shown previously for the GLAD and LASER data that the impacts of inhomogeneity and anisotropy are relatively weak and do not affect the zeroth-order results (*32*, *49*).

Surface drifters have a tendency to cluster into convergent regions (*38*, *42*), which can result in biased sampling. In the present study, this may result in overestimating the KE transfer rates relative to the forward cascade developing at scales smaller than O(1 to 10 km), as dissipative processes are likely to strengthen at the locations where convergent fronts occur (*50*, *51*). In (*43*), analyses performed on subsets of the dataset used here suggested that the Lagrangian drifter–based estimates of quantities stemming from SF3 may be up to three to five times larger than those obtained from the underlying Eulerian fields. In addition, Berta *et al*. (*34*) showed that SF3 estimates differ between different flow features, highlighting the spatial intermittency of the forward cascade. However, despite these sampling and regional differences, both these studies showed that, qualitatively, SF3 is almost always negative at scales smaller than O(1 km). Thus, we acknowledge that, while intrinsic biases in observations prevent us from assessing with very high accuracy the spatially averaged forward KE cascade rate, these do not prevent us from drawing conclusions on the direction of the KE cascade. In addition, our error estimation method already accounts for some of these biases (detailed in the Supplementary Materials). We generated random sample sets from the available data to estimate confidence intervals, and some of these sets are probably less biased than others. Hence, it is likely that the true Eulerian estimates lie within the range of our error bars, which are relatively large but unambiguously signal at the presence of a forward and inverse energy cascade (e.g., Table 1). Currently, no established methods to systematically account for or correct for these sampling biases are available, and this remains an area of active research. In the future, a combination of improved theoretical and statistical tools and better resolved observations and simulations will likely refine our results and conclusions.

Despite these caveats, direct observational evidence of the dual KE cascade is a crucial step forward in our understanding of how oceanic turbulence operates and shows that a direct pathway for dissipation of mesoscale KE is present at the surface ocean. The observational evidence provided here is also relevant from a fundamental standpoint and supports a new paradigm in turbulence, that of a dual KE cascade developing in a fully 3D fluid under the influence of rotation and stratification (*23*, *25*). Our results and methods therefore provide important avenues for future research. On the dynamical side, it remains unclear what sets the ratio of the KE that is cascaded downscale versus upscale and how this ratio is seasonally modulated. One possibility is that the downscale KE cascade might be controlled by the strength of the internal waves, as shown in (*24*, *25*). The corresponding mechanisms may involve a direct cascade of only the KE associated with waves to small scales or mechanisms resulting from the interaction of waves with balanced flows such as the spontaneous loss of balance (*52*) or the stimulated loss of balance (*53*–*55*) and the catalytic effect of internal waves (*51*, *56*). A decomposition of the SF3 into contributions from different parts of the flow could be helpful to answer this question. While a pathway for energy dissipation at the surface ocean has been confirmed by the present study, it remains to be understood what the relative strength of this pathway in dissipating energy and closing the ocean’s energy budget is compared to mechanisms that are active in the interior, at land or ice boundaries, or via interaction with surface forcing.

One major advantage of our methodology is that it does not require the observations to be gridded and can easily handle data gaps and nonuniform sampling, which is the case for most observational platforms including satellites. Thus, it is also worth considering how our analysis methods can be expanded to other observational datasets in the future, such as the surface velocities obtained from the Global Drifter Program, velocity estimates that will come from the Surface Water and Ocean Topography satellite and future Wind and Current Missions, or more targeted observations from process studies where buoyancy measurements are made along with velocity and can allow for consideration of the kinetic and potential energy cycles simultaneously.

## METHODS

### The surface drifters

All the data used in this study were collected by observations from surface drifters, which are devices that follow the surface flow in the ocean, whose locations are tracked using the Global Positioning System (GPS). The location information is then used to infer the surface velocities. The drifters used here tracked the flow in the top 60 cm of the surface. Their positions were retrieved at 5-min intervals with a nominal position error of <10 m, and these raw position estimates were processed and provided by the Consortium for Advanced Research on Transport of Hydrocarbon in the Environment (CARTHE) as processed drifter trajectories, which were low-pass–filtered with a 1-hour cutoff and resampled to a uniform time grid of 15 min. Here, we used drifters that were deployed in two targeted studies in the northeastern GoM.

The GLAD experiment was conducted in the wake of the Deepwater Horizon oil spill. Two hundred ninety-seven coastal dynamics experiment (CODE) style surface drifters (*57*) were released over the period of 11 days at the end of July 2012, making it the largest drifter release experiment at that time (*37*). The trajectories span the period from July to October 2012, which are the summer months.

The LASER experiment was conducted at approximately the same location as the GLAD experiment. A total of approximately 1000 surface drifters were released near the end of January 2016, making it the largest drifter deployment to date. The surface drifters used in LASER, CARTHE style drifters, had a slightly different design than the GLAD drifters; the new design was meant to be more rapidly deployable and also more environmentally friendly (*36*). Despite the design differences, the GLAD and LASER drifters showed similar characteristics at following the flow and, for all practical purposes, are considered to be the same. The LASER trajectories span the period from January to March 2016, which are the winter months. Many of the LASER drifters lost their drogue at some point after their deployment, and we only use the portion of the trajectories when the drogue was attached (*58*).

The drifters were released in clusters, and so, most of the observations at the smallest separation scales (<10 days) are for the duration of late July and early August in GLAD and late January and early February in LASER. The deployments were also often targeted on particular flow features, so the samples at the smallest scales might not be as representative of the true statistics, as the samples gathered once the drifters disperse and randomly sample many different flow features. Even at longer times, the sampling of the drifters cannot be considered to be perfectly comparable to an Eulerian grid because of the horizontal flow following the nature of the sampling, which can result in the drifters spending less time in fast-moving flows relative to the slow-moving flows.

### Statistical metrics and error estimates

The metrics of interest in this study take the general form ⟨δ*u^n^*⟩(*r*), where the ⟨. ⟩ indicates an ensemble averaging operation and δ*u*(*r*) is the difference in a particular velocity component between two points that are separated by distance *r*, and this velocity difference is raised to some power of *n* corresponding to different orders of the structure functions. When estimating these metrics using drifters, we assume that the velocity measured by a particular drifter is the velocity of the surface ocean at that location. Using any two drifters, we get one sample estimate of δ*u*(*r*). Because we want to estimate the metric as a function of separation scale, we divided the separation axis, *r*, into bins that are logarithmically distributed between 10 m and 1000 km using the formula *r_n_* = *r*_0_ × 1. 5*^n^*, where *r*_0_ = 10 m and *n* = (0,1,2,3, …). The ensemble averaging is replaced by averaging over all drifter pairs in any particular bin, which come from different spatial regions and times; this is equivalent to assuming temporal stationarity and spatial homogeneity in a statistical sense. To ensure that the homogeneity assumption is not significantly violated by the sampled flow, we only use pair samples collected by the drifters in a particular region that we expect to have similar dynamics everywhere (fig. S1). We also assume isotropy when averaging over all orientations of the position vectors connecting the two drifters relative to the geographical coordinates.

The error estimates on the metrics are calculated by using a form of bootstrapping called modified block bootstrapping. Regular bootstrapping is done by estimating the same statistical metric multiple times by random sampling with replacement, keeping the number of samples the same as the original sample size, from the observed distribution of the samples [the samples for us are measurements of δ*u*(*r*)*^n^* in some separation bin, where the number of samples is smaller than the number of drifter pairs]. The mean over these multiple estimates of the metrics is then used as the estimate of the metric, and percentiles of these distributions can be used as estimates of the error—here, we use the 5th and 95th percentiles. However, one key assumption in regular bootstrapping is that all the samples are independent, which is not even approximately true for our dataset because of the temporal and spatial correlations between the different pairs. Instead, in block bootstrapping, the dataset is divided into blocks that are approximately independent, and the resampling is done over these blocks rather than over all the individual samples. Here, the blocks were defined by (i) estimating the total duration (*T*_tot_) over which a most of the data was collected, defined approximately as 90 days during GLAD and 60 days during LASER; (ii) estimating the time scale corresponding to each scale [*T*_scale_(*r*)], which was estimated as $T_{\text{scale}}(r)=r/\sqrt{D_{\text{tot}}}$; (iii) defining the number of degrees of freedom as *N*^DOF^ = *T*_tot_/*T*_scale_; and then (iv) dividing the total number of samples in each bin (arranged in order with the *m* individual time series for the *m* pairs that spent some time in the particular separation bin) into *N*^DOF^ blocks. This is an approximate but pragmatic procedure, and because of some degree of independence between the different pair time series in each bin, it actually results in an upper bound on the error estimates, because we assume that there are less independent blocks than there actually might be (some independent time series from different pairs might end up in the same block when using our algorithm). This method is described further in section SB.

### SF2 as proxy for scale-wise KE distribution

The longitudinal and transverse components of the SF2 are defined as

$$D_{LL}(r)={\left(\delta u_{L}\right)}^{2},\;D_{TT}(r)={\left(\delta u_{T}\right)}^{2}$$ (1)

where δ*u_L_* and δ*u_T_* are longitudinal and transverse velocity differences, respectively. These are defined as

$$\delta u_{L}=\delta u\cdot \frac{r}{|r|}\text{and}\;\delta u_{T}=\delta u\cdot t$$ (2)

where **r** is the vector connecting the two points (**x**_1_ and **x**_2_), where simultaneous velocity observations are made (**r** = **x**_2_ − **x**_1_), and **t** is the unit vector perpendicular to it (**r** · **t** = 0) on the horizontal plane. δ**u** = **u**_2_ − **u**_1_ is the difference in velocity between the two points. We refer to the sum of these two components as the total SF2 (*D*_tot_ = *D_LL_* + *D_TT_*). Plots of the components are presented in the Supplementary Materials (fig. S5).

The SF2 is related to the corresponding KE as an integral relationship, $D_{i}(r)=\int_{0}^{\infty }E_{i}(k)(1-J_{0}(kr))dk$, with *i* = *LL* or *TT*, which is a result of Fourier transformation of isotropic 2D fields. Here, *E_i_*(*k*) is the longitudinal or transverse component of the KE, and *J*_0_(*x*) is the zeroth-order Bessel function [further discussion of this relationship can be found in (*32*)]. Roughly, the SF2 behaves similar to the cumulative sum of KE up to a particular scale when the spectral slope of KE is shallower than *k*^−3^, and in these regimes, larger values of SF2 at any scale usually correspond to larger values of KE near that scale. Thus, the SF2 is not precisely related to the KE at a particular scale but is rather a rough proxy. Unfortunately, inverting the relationship to estimate the KE from the SF2 results in an incoherent solution due to amplification of noise and is therefore not attempted here.

However, we confirmed that the SF2 behaves as a rough proxy for KE distribution by using an alternate metric of the distribution of KE as a function of scale, called the signature function (section SD) (*59*), and by testing the relationship between the SF2 and KE using idealized functional forms with known KE distribution.

The longitudinal and transverse components can be used to infer the rotational (*D*_R_) and divergent (*D*_D_) contributions to total SF2, using a Helmholtz decomposition (*41*, *60*). This is performed by using the formulae

$$D_{R}(r)=D_{TT}(r)+\int_{0}^{\infty }\frac{1}{r}(D_{TT}(r)-D_{LL}(r))dr$$ (3)

$$D_{D}(r)=D_{LL}(r)-\int_{0}^{\infty }\frac{1}{r}(D_{TT}(r)-D_{LL}(r))dr$$ (4)

The rotational and divergent estimates are more useful as they are closely related to different dynamical regimes, where geostrophically balanced vortical flows are primarily rotational in nature, while ageostrohic flows or internal waves have a major contribution from the divergent part. These formulae are derived under assumptions of isotropy and homogeneity. When these assumptions are not perfectly satisfied, the estimates can have some nonphysical characteristics. In particular, at scales where one component (rotational or divergent) is significantly smaller than the other, the formulae can result in negative values for the SF2 (shown in fig. S6 and can be seen as a range of scales—larger than 500 km in summer and between 500 m and 50 km in winter—where SF2_D_ is not plotted in Fig. 2).

Comparable values at the largest scales (scales larger than 200 km in winter) are likely a result of breakdown of the assumptions of homogeneity and isotropy. We do not expect these issues to qualitatively alter the results presented here (*49*). Some corrections can be introduced to account for factors such as anisotropy, e.g., (*49*), but were not used here because, for the particular datasets under consideration, the added complexity does not affect the qualitative conclusions.

### SF3 and interscale energy transfers

The SF3 [or *V*(*r*)], defined as

$$V(r)=\langle \delta u_{L}(\delta u_{L}^{2}+\delta u_{T}^{2})\rangle$$ (5)

is related to the KE spectral flux [*F*(*k*)] through a Fourier transform as

$$V(r)=-4r\int_{0}^{\infty }\frac{1}{k}F(k)J_{2}(kr)dk$$ (6)

where *J*_2_(*x*) is the second-order Bessel function.

Here, we discretize *F*(*k*) using a set of piecewise constant basis

$$F(k)=-\epsilon_{u}+\sum_{j=1}^{N_{f}}\epsilon_{j}H(k-k_{j}){dk}_{j}$$ (7)

where ϵ*_u_* is the upscale KE transfer rate (units L^2^/T^3^) and ϵ*_j_* is the KE injection density (KE injection per unit wave number; units L/T^3^) at wave number *k_j_*. The total KE input into the system would be ∑*_j_*ϵ*_j_dk_j_*, and this sum can be estimated over a fixed range of scales to estimate the KE injection (units L^2^/T^3^) at those scales (as done for Table 1).

Substituting Eq. 7 into Eq. 6, we obtain

$$V(r)=2\epsilon_{u}r-\sum_{j=1}^{N_{f}}\;4\frac{\epsilon_{j}}{k_{j}}J_{1}({rk}_{j}){dk}_{j}$$ (8)

which links the measurable SF3 with physically important quantities including the KE injection rates at different scales and the upscale KE transfer rate.

In this study, we estimate *V*(*r*) from surface drifter data and fit this using Eq. 8 to obtain ϵ*_u_* and ϵ*_j_*, which are then be used to gain insights into the KE transfer across scales, e.g., by calculating the corresponding *F*(*k*) using Eq. 7. We fit the estimated *V*(*r*) over the range of scales from 50 m to 500 km to avoid scales where the estimates of *V*(*r*) are less certain. However, the exact choice of this range does not affect the results of our study. The details of this estimation problem (inverse problem) and the relationship between the spectral flux and SF3 are discussed in section SF.

## Supplementary Materials

### This PDF file includes:

Sections SA to SG Figs. S1 to S9
