# Supplementary material for: Direct observational evidence of an oceanic dual kinetic energy cascade and its seasonality
Source: Sci Adv. 2022 Oct 12;8(41):eabq2566. doi: 10.1126/sciadv.abq2566 (PMC9555769; doi:10.1126/sciadv.abq2566)
Supplement: Supplementary file 1 — Sections SA to SG Figs. S1 to S9 [file sciadv.abq2566_sm.pdf]

Science Advances
AAAS

Supplementary Materials for

Direct observational evidence of an oceanic dual kinetic energy cascade and its seasonality

Dhruv Balwada *et al.*

Corresponding author: Jin-Han Xie, jinhanxie@pku.edu.cn

Sci. Adv. **8**, eabq2566 (2022)
DOI: 10.1126/sciadv.abq2566

This PDF file includes:

Sections SA to SG
Figs. S1 to S9

Supplementary Information

A GLAD and LASER data distribution

Figure S1 shows the distribution of data as a function of separation scale, time, and spatial location. A subset of the full data set (Figure S2) was chosen for the analysis, the region defined between 91W and 84W, North of 24N, and in ocean deeper than 500 m, to ensure near homogeneity and similar dynamics over the samples. Many drifters during the LASER experiment lost their drogues (undrogued trajectories are not used in this study), which leads to a rapid decrease of observed pairs in Figure S1e, even though approximately 3 times as many drifters as the GLAD experiment were deployed.

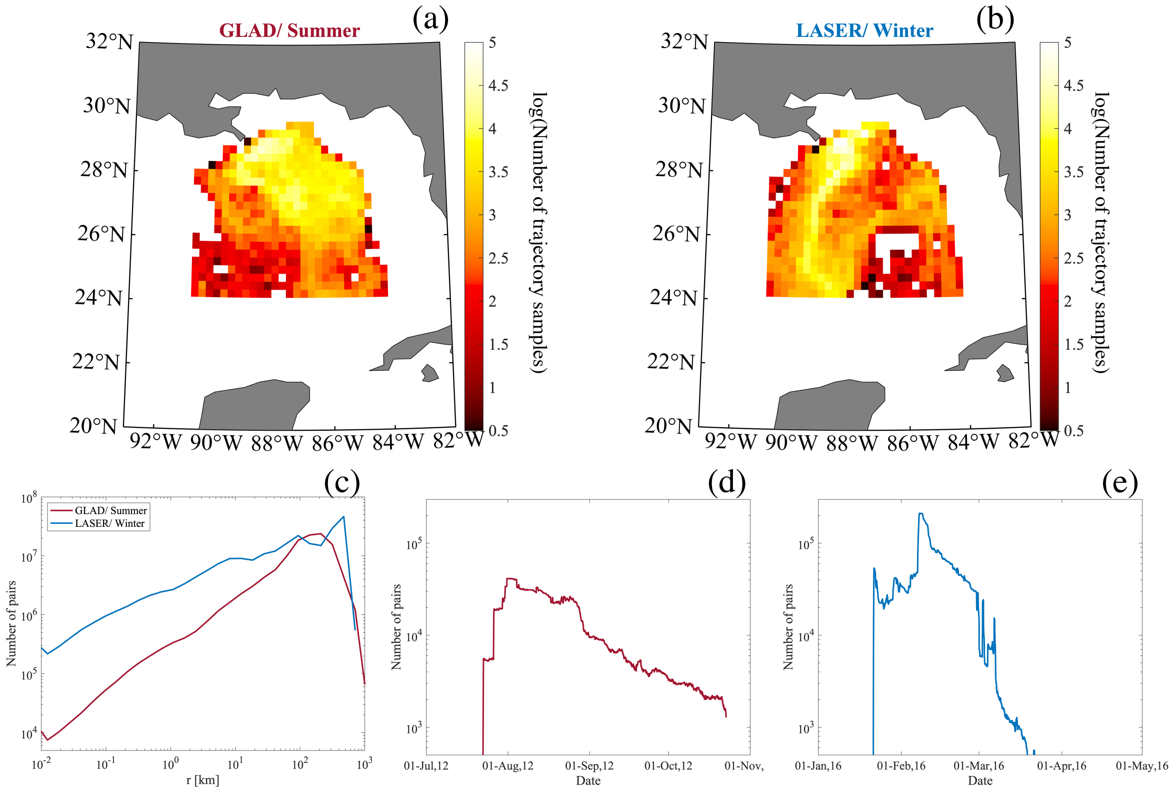

Figure S1: **Data distribution** (a,b) Number of trajectory samples in $0.25^o \times 0.25^o$ bins over the region that was used for the analysis (91W to 84W, North of 24N, and in ocean deeper than 500 m), (c) number of pairs as per separation, (d, e) number of pairs as a function of time for GLAD and LASER respectively.

B Error estimates - Modified block bootstrapping

In this work we need to estimate errors or confidence intervals on the sample mean of a complex statistic $\delta u(r)^n$. Estimating these errors is non-trivial because no analytical form is known for the sample distribution (distribution of δu^n, Figure S2), and no standard formula for the error estimate is available. So here we use a variant of the more general error estimation technique known as bootstrapping (`https://en.wikipedia.org/wiki/Bootstrapping_(statistics)`), which involves random resampling with repetitions from the available data samples (mimicking the sampling process) and estimating the means using these randomly generated datasets. The

distribution of these estimated means can then be used to infer the errorbars or the confidence intervals.

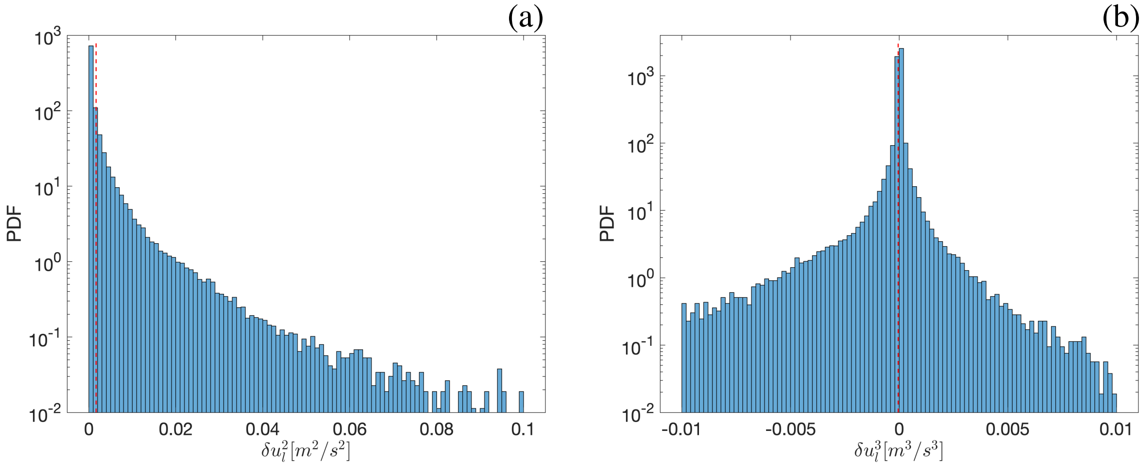

Figure S2: Distribution of $\delta u^2(r)$ (a) and $\delta u^3(r)$ for the GLAD experiment for the bin between 577 and 865m. The sample means are shown as the vertical red lines.

This standard bootstrapping technique is not appropriate for us because it assumes that all available samples are independent, which is not the case here. Our samples are collected by pairs of surface drifters that are within a separation distance belonging to a finite-sized separation bin. Since the drifter pair can potentially stay in a particular separation bin for a finite amount of time, particularly in the larger bins at greater separations, we have time series that are temporally correlated (Figure S3). Additionally, if two or more pairs are in close proximity to each other and sampling the same flow feature the samples collected by the different pairs will also have some spatial correlation. The Lagrangian nature of the sampling mixes these temporal and spatial correlations.

Since the samples are correlated we looked towards *block bootstrapping*. This technique is usually used with time-series data, where the total time series is first divided into blocks based on some temporal correlation scale. For example, if the correlation scale is 10 days and the time series is 100 days long we would divide the time series into ten 10 day blocks. The resampling

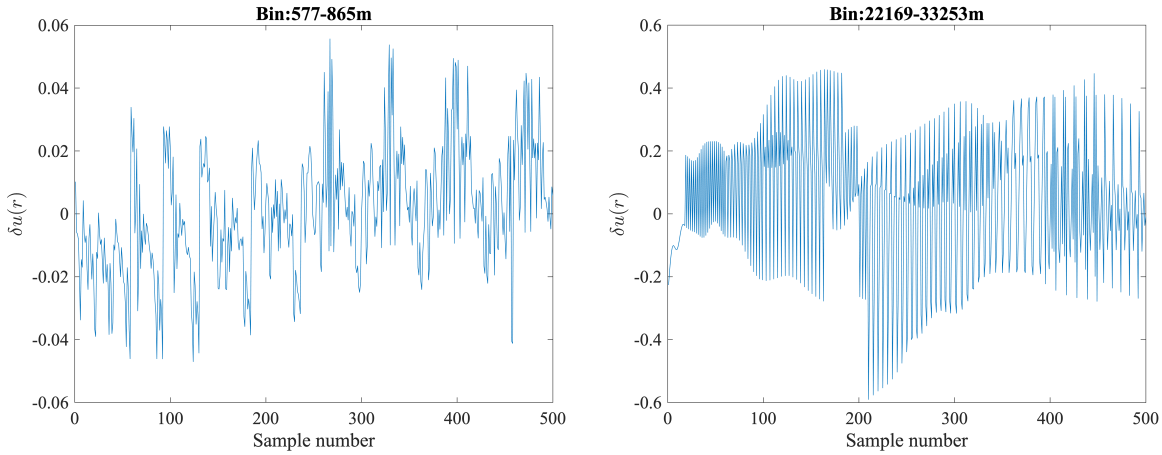

Figure S3: δu obtained from samples in two different separation bins - showing temporal coherence, and hence a lack of independence between samples.

is then done using these blocks as individual samples. Here we do not have a single time series but rather a concatenated time series made up of different pairs in a separation bin (Figure S3), where the different pairs are also likely to be correlated due to spatial proximity – particularly at large-separation scales that are accounting for larger flow features that evolve slowly. Thus, using the traditional block bootstrapping also does not work for us; we experimented with this and found the error estimates to be intuitively too small (not shown).

Instead we use a modified version of the block bootstrapping approach. We first estimate the possible degrees of freedom for a particular separation bin based on a temporal correlation scale and the total duration of the experiment. The temporal correlation scale was estimated using the estimate of the total SF2, as $T_{scale}(r) = r/\sqrt{D_{tot}(r)}$. The duration of the experiment, T_{total} was chosen as roughly the duration over which a large number of pairs are present, and was chosen as 90 days for GLAD and 60 days for LASER (Figure S1d,e). Then the number of degrees of freedom was defined as $N^{DOF}(r) = T_{total}/T_{scale}(r)$, and is shown in Figure S4 for the two experiments. As one might expect the $N^{DOF}(r)$ decreases as a function of scale, since fewer independent large-scale events are sampled. Then we took the concatenated set of

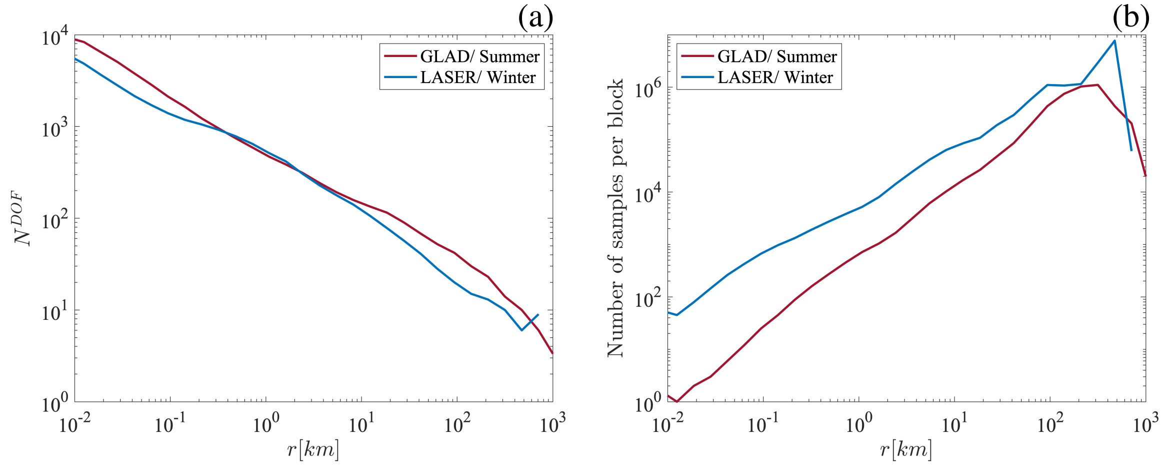

Figure S4: (a) Number of degrees of freedom (same as number of blocks) as a function of separation scale, and (b) number of samples per block as a function of separation scale.

samples for any separation bin and divided them into $N_{DOF}(r)$ blocks, and used these blocks as independent samples for block bootstrapping. Our underestimate of $N_{DOF}(r)$ is most likely smaller than the actually degrees of freedom (which is unknown), since we are not accounting for the fact that some samples might be uncorrelated because of large spatial separation, and is likely to give an upper bound on the error estimates. Our approach to estimating the blocks and consequently the errors is approximate but pragmatic, and we hope that future work can develop more precise error estimates.

C Additional plots of 2nd-order structure functions

Figure S5 shows the two raw components, longitudinal and transverse, of the total SF2 and figure S6 shows the two decomposed components, rotational and divergent, of the total SF2. The information in figure S6 is the same as Figure 2, and is shown on a linear axis so that the negative values can be seen. In principle all the components of the SF2 should be positive, but the divergent components takes negative values over some range of scales. These negative values are small compared to the rotational or total components, about 20% at maximum but

usually much smaller contribution to the total. This non-physical result is likely because the assumptions required to derive the structure function decomposition formulae (equation 3 and 4) are not perfectly satisfied, and this failure in the method has been discussed in length in (*49*).

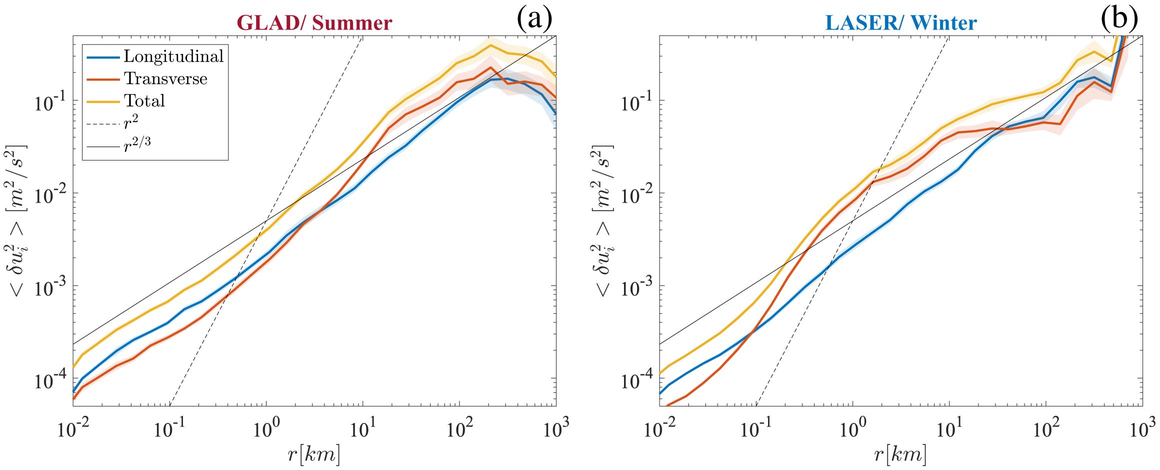

Figure S5: Longitudinal (D_{LL}) and transverse (D_{TT}) components of SF2 for the (a) GLAD and (b) LASER experiments.

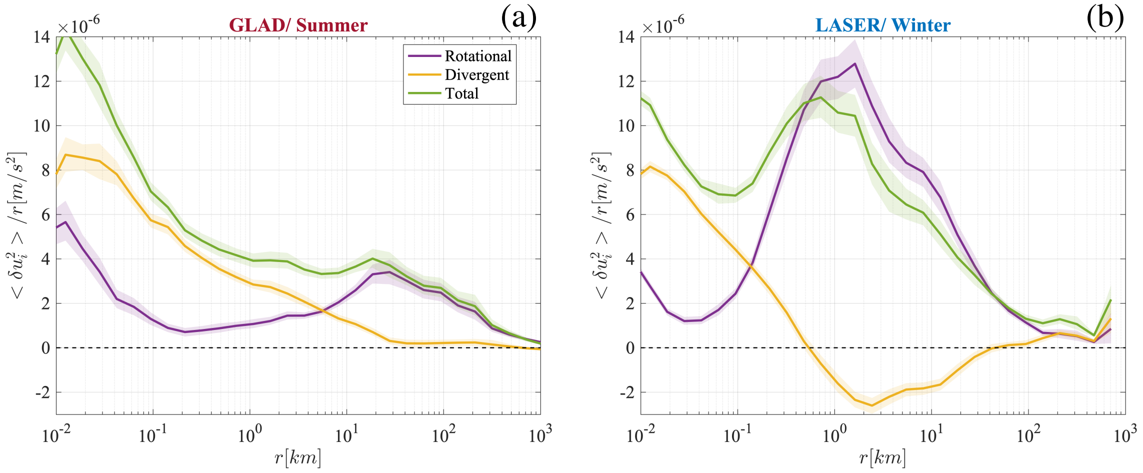

Figure S6: Rotational (D_R) and divergent (D_R) components of SF2 for the (a) GLAD and (b) LASER experiments compensated by r for visualization purpose on a linear axis.

D Signature functions - an alternate metric for distribution of kinetic energy as a function of scale

SF2 represent a smoothed and cumulative distribution of KE across scales. This is because at any scale the SF2 includes a sum of energy from all smaller scales, but also enstrophy from all larger scales (which is often small as enstrophy is usually cascading downscale) (*32, 59*). (*59*) proposed signature function as an alternative metric that is better representative of the KE at different scales, and can almost be considered comparable to the KE spectrum. It is defined as,

$$G(r) = -\frac{r^2}{4}\frac{\partial}{\partial r}\left(\frac{1}{r}\frac{\partial}{\partial r}\left\langle \delta u^2 \right\rangle\right). \tag{S1}$$

One caveat of the signature function when working with observational estimates is the requirement to estimate derivatives. Here we ameliorate the noisy nature of derivatives by fitting a 5th-order polynomial to the SF2 before estimating the signature function (the results here are not overly sensitive to this choice).

The signature function estimates are shown in Figure S7. While there are some quantitative differences compared to the SF2, in part due to the polynomial fitting, none of the major qualitative results about the relative behavior of winter vs summer or rotational vs divergent flows change. Part of the reason for this is that most of the enstrophy in the type of flows we are considering is expected to be at the smaller scales, and so the SF2 is a good metric to express the distribution of KE across scales. It is also interesting to note that the signature functions peak around a scale of 100km, the dominant energy containing scale in the ocean, which is because signature functions are a measure of KE per scale rather than a cumulative metric like SF2.

E Details of the 3rd-order structure function theory

Here we first discuss the derivation of the Karman-Howarth-Monin (KHM) equation in our context, and then discuss the relationship between the third-order structure function and the

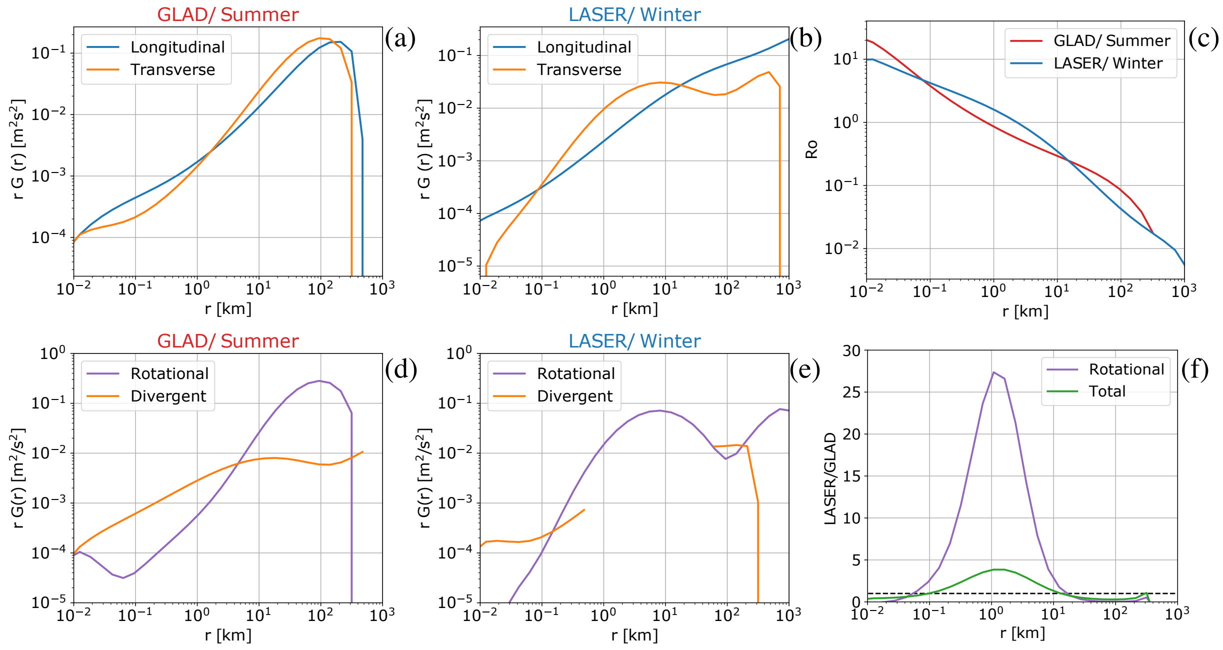

Figure S7: **Signature functions for the GLAD and LASER experiment.** (a, b) The longitudinal and transverse components. (c) the Rossby number for the two experiments. (d, e) The rotational and divergent decomposition. (f) The ratio of the total and rotational components between the two experiments.

spectral flux.

E.1 Derivation of the KHM equation

The goal of the theory is to describe the dynamics influencing the horizontal velocity correlations (which are related to the SF2 and correspondingly the KE), and how the non-linear interactions leading to cross-scale energy transfers can be quantified by using the 3rd-order structure functions. We focus on horizontal KE, since in the ocean at the scales of interest the the horizontal KE is the dominant reservoir of KE, and also because the drifters only track the horizontal flow.

We consider the f-plane horizontal momentum equation,

$$\boldsymbol{u}_t + \nabla \cdot (\boldsymbol{u}\boldsymbol{u}) + \partial_z (w\boldsymbol{u}) + f\boldsymbol{u}^{\perp} = -\nabla p + \boldsymbol{F} + \boldsymbol{D}, \tag{S2}$$

where $\boldsymbol{u} = (u, v)$ is the horizontal velocity, $\boldsymbol{u}^{\perp} = (-v, u)$, w is the vertical velocity, $\nabla = (\partial_x, \partial_y)$ is the horizontal gradient, f is the Coriolis frequency, p is the pressure, $\boldsymbol{F}$ is an external force, and $\boldsymbol{D}$ denotes the dissipation. The dissipation term can contain both large- and small-scale dissipation.

To derive expressions for quantities that can be estimated using horizontal movement of drifters, we next consider two-point correlations with horizontal displacements. We denote the horizontal locations of two points as $\boldsymbol{x}_1$ and $\boldsymbol{x}_2$, respectively, and $\boldsymbol{r} = \boldsymbol{x}_2 - \boldsymbol{x}_1$ is the horizontal displacement vector between these two points. Thus, we define the velocity at location $\boldsymbol{x}_i$ as $\boldsymbol{u}_i$, and the velocity difference is defined as

$$\delta\boldsymbol{u} = \boldsymbol{u}_2 - \boldsymbol{u}_1. \tag{S3}$$

By assuming *horizontal homogeneity* we obtain the following relation between the spatial derivatives (*26*),

$$\nabla \equiv \nabla_{\boldsymbol{r}} = \nabla_{\boldsymbol{x}_2} = -\nabla_{\boldsymbol{x}_1}. \tag{S4}$$

Homogeneity also results in the statistics of velocity difference to only be a function of the displacement. e.g.

$$\overline{\delta \boldsymbol{u}^2}(\boldsymbol{x}_1, \boldsymbol{x}_2) = \overline{\delta \boldsymbol{u}^2}(\boldsymbol{r}), \tag{S5}$$

where the $\overline{(.)}$ denotes an ensemble average. Practically the ensemble average is approximated by a time and space average over all drifter pairs (discussed in "Statistical metrics and error estimates" in the Methods). The assumption of homogeneity is foremost a pragmatic one, since it allows us to average over the full data set to ensure robust statistics.

Multiplying $\boldsymbol{u}_1$ to the momentum equation (S2) evaluated at $\boldsymbol{x}_2$, adding the conjugate equation, and then assuming a statistically steady state, we obtain the steady KHM equation (*26*),

$$-\frac{1}{4}\nabla \cdot \boldsymbol{V} = P + D, \tag{S6}$$

where

$$\boldsymbol{V} = \overline{\delta \boldsymbol{u} |\delta \boldsymbol{u}|^2}, \tag{S7a}$$

$$P = \frac{1}{4}\nabla \cdot \left(\overline{\boldsymbol{u}_2 |\boldsymbol{u}_1|^2} - \overline{\boldsymbol{u}_1 |\boldsymbol{u}_2|^2} \right) - \frac{1}{2}\left(\overline{\boldsymbol{u}_1 \cdot \partial_z \left(w_2 \boldsymbol{u}_2 \right)} + \overline{\boldsymbol{u}_2 \cdot \partial_z \left(w_1 \boldsymbol{u}_1 \right)} \right)$$
$$+ \frac{1}{2}\nabla \cdot \left(\overline{\boldsymbol{u}_2 p_1} - \overline{\boldsymbol{u}_1 p_2} \right) + \frac{1}{2}\left(\overline{\boldsymbol{u}_1 \cdot \boldsymbol{F}_2} + \overline{\boldsymbol{u}_2 \cdot \boldsymbol{F}_1} \right) \tag{S7b}$$

$$D = \frac{1}{2}\left(\overline{\boldsymbol{u}_1 \cdot \boldsymbol{D}_2} + \overline{\boldsymbol{u}_2 \cdot \boldsymbol{D}_1} \right). \tag{S7c}$$

$\boldsymbol{V}$ is the 3rd-order structure-function vector. P subsumes terms corresponding to nonzero horizontal divergence, vertical velocity, pressure gradient and the external-forcing. D is the effect of dissipation.

If we further assume hydrostatic balance in the vertical and vertical homogeneity we can express the pressure-related term in (S7b) through buoyancy θ as

$$\nabla \cdot \left(\overline{\boldsymbol{u}_2 p_1} - \overline{\boldsymbol{u}_1 p_2} \right) = \overline{w_1 \theta_2} + \overline{w_2 \theta_1}, \tag{S8}$$

which corresponds to conversion between potential and KE through processes such as baroclinic instability.

Further, we also assume *isotropy*. (*32, 49*) showed that this assumption is not strictly valid for the datasets under consideration, but it serves as a pragmatic assumption. Also, we average measured velocity differences in different directions with the same two-point distance (r), which is equivalent to an azimuthal/angle average that removes the dependence on orientation or angle. If the displacements of two measured points are uniformly distributed with azimuthal angle, this average procedure keeps the isotropic components and therefore the isotropic theory works. Nuances associated with the assumptions of homogeneity and isotropy can be explored in future work.

Thus,

$$\boldsymbol{V} = V(r)\boldsymbol{e}_r, \tag{S9}$$

where $\boldsymbol{e}_r = (x/r,\, y/r)$ with $r = \sqrt{x^2 + y^2}$ and $\boldsymbol{e}_r$ is an unit vector. We can estimate V directly from the velocity measured by the drifters,

$$V(r) = \overline{\delta u_L\,(\delta u_L^2 + \delta u_T^2)}, \tag{S10}$$

where δu_L and δu_T are longitudinal and transversal velocity differences, respectively. And they are defined as

$$\delta u_L = \delta \boldsymbol{u} \cdot \frac{\boldsymbol{r}}{|\boldsymbol{r}|} \quad \text{and} \quad \delta u_T = \delta \boldsymbol{u} \cdot \boldsymbol{t}, \tag{S11}$$

where the unit vector $\boldsymbol{t}$ satisfies $\boldsymbol{t} \cdot \boldsymbol{r} = 0$ and $\boldsymbol{r} \times \boldsymbol{t} = \boldsymbol{z}$ with $\boldsymbol{z}$ the vertical unit vector.

E.2 Relationship between the 3rd-order structure function and spectral flux

We showed above that the the SF3 or $V(r)$ is present as a term in the KHM equation, which describes the dynamics of two-point velocity correlations. An analogous equation to the KHM equation can also be derived in spectral space, which describes the dynamics of the velocity power spectra (*26*).

Note that the two-point correlation and power spectra are related via a Fourier transform. Thus, the third-order structure function can be related to the spectral flux via an integral relationship,

$$V(r) = -4r \int_0^\infty \frac{1}{K} F(K) J_2(Kr) dK, \tag{S12}$$

where J_2 is the second-order Bessel function. The Bessel function emerges from a Fourier transform due to the assumption of isotropy (*35*).

Case of a single injection scale

A common ideal scenario that is considered in turbulence theories is an energy injection at a single forcing scale (k_f), which corresponds to the spectral flux

$$F(K) = -\epsilon_u + \epsilon H(k - k_f), \tag{S13}$$

where ϵ_u is the energy flux upscale and ϵ is the energy injection at the scale corresponding to k_f. This expression assumes that some fraction of the injected energy is fluxed upscale and the rest downscale, here the magnitude of downscale energy flux is $\epsilon_\mathrm{d} = \epsilon - \epsilon_\mathrm{u}$. Here it is assumed that the dissipation only happens at zero and infinite wavenumbers.

Substituting (S13) into (S12) results in the corresponding expression of the SF3 (*35*):

$$V(r) = 2\epsilon_\mathrm{u} r - 4\epsilon k_f J_1(k_f r), \tag{S14}$$

where J_1 is the Bessel function of the first order.

Expression (S14) has three power-law ranges, corresponding to the inertial ranges of upscale energy transfer, downscale energy transfer and downscale enstrophy transfer, respectively.

These ranges are shown in the asymptotic expansions as follows

$$V(r) = \begin{cases} \underbrace{-2\epsilon_{\mathrm{d}} r}_{\text{downscale energy}} + \underbrace{\frac{1}{4}\left(\frac{r}{l_f}\right)^2 \epsilon r}_{\text{"enstrophy"}} + O\left(\left(\frac{r}{l_f}\right)^5\right), & \text{when} \quad \frac{r}{l_f} \ll 1, \\ \underbrace{2\epsilon_u r}_{\text{upscale energy}} + O\left(\left(\frac{r}{l_f}\right)^{-1/2}\right), & \text{when} \quad \frac{r}{l_f} \gg 1. \end{cases} \tag{S15}$$

By capturing the three inertial ranges in one formula with resolved forcing scale and with the applicability to the scenario of bidirectional energy transfer, expression (S14) solves shortcomings of the previous theories which are only applicable to inertial ranges with unidirectional energy transfer, and therefore it provides the foundation of analyzing third-order structure function measured in geophysical turbulence. Another advantage of the new theory is that it can be used to detect the energy injection scales and the magnitude of energy injection at each scale, which was not possible using classic inertial-range theories because inertial range by definition is away from the forcing scales. Notice that if there is no downscale flux of energy ($\epsilon_d = 0$), as is the case in the classical 2D turbulence paradigm, we will only observe a signature of the enstrophy range at scales smaller than l_f.

Enstrophy is an important quantity in geophysical turbulence, especially in the quasigeostrophic approximation. As a quasi-two-dimensional flow, we can define enstrophy as ω^2 with vorticity $\omega = v_x - u_y$, and the third-order structure function corresponding to the enstrophy flux (derived by considering the two point vorticity correlation equation) is

$$\boldsymbol{V}_\omega = \overline{\delta \boldsymbol{u} \delta \omega^2}, \tag{S16}$$

under the assumption of isotropy we have $\boldsymbol{V}_\omega = V_\omega(r)\boldsymbol{e}_r$. From the perspective of KHM equation, the enstrophy and energy third-order structure functions are linked by a Laplacian

$$\nabla \cdot \boldsymbol{V}_\omega = -\nabla^2 \nabla \cdot \boldsymbol{V}, \tag{S17}$$

Therefore, for the isotropic energy structure-function expression (S14) with bidirectional energy flux, the corresponding enstrophy structure function is

$$V_\omega(r) = -4\epsilon k_f^3 J_1(k_f r), \tag{S18}$$

which implies that an enstrophy flux from wavenumber k_f to small scales with a strength of ϵk_f^2. The associated spectral flux of enstrophy is

$$F_\omega(k) = \epsilon k_f^2 H(k - k_f). \tag{S19}$$

Thus, the energy structure functions embed all the information contained in the enstrophy structure function, therefore here the enstrophy structure function is not directly addressed. Additionally, once we have estimated the ϵs from the procedure described next, we could look at the rates of enstrophy fluxes if needed.

F Details of parameter estimation method

F.1 Formulation of the discrete problem

Gaining inspiration from equation (S13), we can express any general form of the spectral flux as

$$F(k) = -\epsilon_u + \sum_{j=1}^{N_f} \epsilon_j H(k - k_j) dk_j, \tag{S20}$$

where ϵ_u is the upscale energy transfer rate (units L^2/T^3), ϵ_j is the energy injection density at scale k_j (energy injection per unit wavenumber, units L/T^3). Note that we indexed dk_j to denote that the forcing wavenumber spacing does not need to be regular. This equation is a discrete representation of the true spectral flux using a set of piece-wise constant basis function.

The equation above can then be passed through the same procedure as for the single forcing scale (equation S14), to derive the corresponding expression for the SF3,

$$V(r) = 2\epsilon_{\mathrm{u}} r - \sum_{j=1}^{N_f} 4\frac{\epsilon_j}{k_j} J_1(r k_j) dk_j. \tag{S21}$$

We fit the observational estimate of SF3 using this expression to estimate the parameters, and hence obtain an estimate of the corresponding spectral flux. We do not directly use (equation 6) to obtain energy flux from the observed third-order structure functions to avoid the amplification of small-scale error in the inverse problem.

The V is estimated at discrete scales r_i with $i = 1, 2, ..., N_r$, which are set based on the used binning. Thus, we obtain a linear equation for ϵ_u and $\epsilon_j (= \epsilon_f(k_j))$,

$$\begin{bmatrix} V(r_1) \\ V(r_2) \\ \cdots \\ V(r_{N_r}) \end{bmatrix} = \begin{bmatrix} 2r_1 & -4\frac{dk_1}{k_1}J_1(r_1k_1) & -4\frac{dk_2}{k_2}J_1(r_1k_2) & ... & -4\frac{dk_{N_f}}{k_{N_f}}J_1(r_1k_{N_f}) \\ 2r_2 & -4\frac{dk_1}{k_1}J_1(r_2k_1) & -4\frac{dk_2}{k_2}J_1(r_2k_2) & ... & -4\frac{dk_{N_f}}{k_{N_f}}J_1(r_2k_{N_f}) \\ \cdots\cdots & & & & \\ 2r_{N_r} & -4\frac{dk_1}{k_1}J_1(r_{N_r}k_1) & -4\frac{dk_2}{k_2}J_1(r_{N_r}k_2) & ... & -4\frac{dk_{N_f}}{k_{N_f}}J_1(r_{N_r}k_{N_f}) \end{bmatrix} \begin{bmatrix} \epsilon_u \\ \epsilon_1 \\ \epsilon_2 \\ \cdots \\ \epsilon_{Nf} \end{bmatrix}. \tag{S22}$$

The large matrix on the RHS with the known parameters, based on choice of discretization, has size $N_r \times (N_f + 1)$. Thus the problem becomes one of solving a linear system of equations,

$$\mathbf{Ax} = \mathbf{b}, \tag{S23}$$

where $\mathbf{x}$ is the vector of unknown ϵs, $\mathbf{b}$ is the vector of observed SF3, and the $\mathbf{A}$ is the matrix formed by the particular relationship (equation S22) between them and the choice of discretization. Solving such system of equations is the subject of discrete inverse theory. Since we get to set N_r and N_f we can setup this problem as an under-determined ($N_r < N_f + 1$), even determined ($N_r = N_f + 1$), or over-determined (($N_r > N_f + 1$) problem. Here we decided to take the over-determined route, and find the solution using a form of the least-squares method, so we can deduce a unique solution under the presence of noise.

F.2 Least-squares method (not used for main results)

The least-squares method solves equation (S23) by minimizing,

$$||\mathbf{Ax} - \mathbf{b}||_2^2, \tag{S24}$$

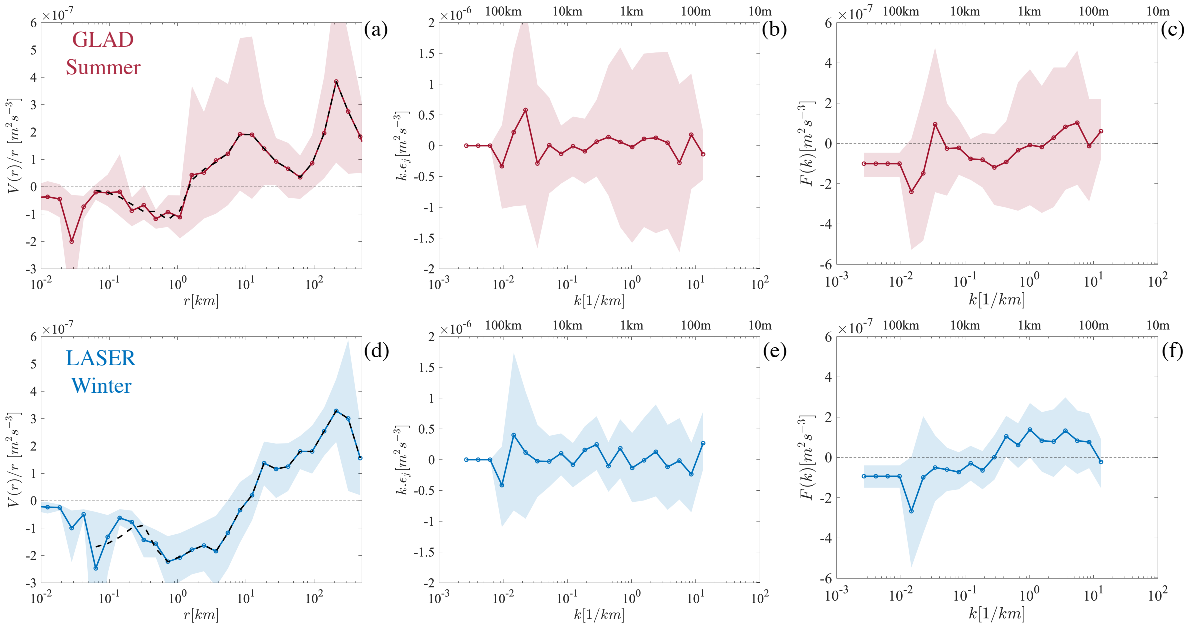

Figure S8: Fits to V(r) (dashed black line in first column) and parameter estimates using the least-square method for the GLAD (top) and the LASER (bottom) experiments. The detailed descriptions of the panels match those of Figure 4. However, notice that the y-axis ranges on the panels b,c,e,f are slightly different.

the L-2 norm (mean square error). If we solve this optimization problem in MATLAB directly using the \ operator, we obtain solutions presented in Figure S8.

The fitted $V(r)$ matches the observed $V(r)$ really well, capturing most of the details. The inferred energy injection is very variable and it is impossible to derive much physical insight from this. The estimated spectral flux is also quite variable, but a rough pattern of downscale flux at smaller scales and upscale flux at larger wavenumbers is suggested.

The problem with this solution is that the optimization method has over-fit the data, providing a really good fit to every detail but little insight. This problem is addressed by using regularization, where some penalty term is added to equation (S24) to impose some additional physical constraints (like smoothness). The regularized solution can be designed to not fit all the stochastic variability in the observations, but instead only the broader pattern that might be

more physically interpretable. One particular regularization approach that we used is discussed next.

F.3 Non-negative least-squares method (used for the main results)

Here we use a particular type of regularization where it is assumed that all the parameters to be estimated are non-negative ($\mathbf{x} \geq 0$). Thus, we assumed that ϵ_u and ϵ_j are all positive, and used the function *lsqnonneg* in MATLAB to solve the system (S23). This assumption is equivalent to assuming that the spectral flux, F, is an increasing function. This is physically justified because we expect there to be an inverse cascade at smaller wavenumbers followed by a forward cascade at larger wavenumbers, and also expect the dissipation to take place at scales outside the observed range. The one downside of this assumption is there is some sink of KE over the fitted scales, like conversion of surface KE to potential energy, it will be artificially smoothed over. However as shown in the previous section, some regularization is necessary to derive more physical insight and so we chose this pragmatic approach. The solution from this method is shown in Figure 4 and discussed in the main text. In future work other regularization methods, like constraints only on smoothness, can be tried in the fitting procedure.

G Application to a simulation of rotating stratified turbulence

To show the efficacy of the above methodology at detecting the KE injection scales and the spectral flux, we applied it to a direct numerical simulation of rotating stratified turbulence. The numerical simulation is of a 3D triply-periodic incompressible Boussinesq equations, and were presented in (*25*). A constant background stratification is prescribed and the external mechanical forcing is isotropic and generated randomly, applied in a shell of modes with wave numbers ($k_f = 10, 11$).

This simulation was selected because it has the dynamical components that we expect to see in the ocean, we have precise knowledge of the energy injection, and a large range of scales smaller than the forcing scale are resolved and simulate a downscale KE transfers. Also, more practically the qualitative structure of the V is similar to what is observed and shown in Figure 3; V is negative at smaller scales and positive at larger scales, and its absolute value approximately follows a linear power law (Figure S9a). We show that equation (S21) can be fit quite well (red line in Figure S9a) to the model V, by optimizing the free parameters: the KE injection rates and scales (Figure S9b) and the upscale KE flux.

Furthermore, using the detected KE injection rate and the upscale KE flux we can reconstruct the KE flux in the spectral space, which is compared with that obtained directly from the numerical simulation in Figure S9c. Notice that the fitting only approximately matches the numerical $V(r)$ over the range of scales where it is negative, this is because $F(k)$ is not a perfectly monotonically increasing function. Implying that some small negative values of ϵ_j would be needed for a better fit, as should be expected given the slight decrease in F(k) at wavenumbers larger than 10. In fact, these scales are associated with a transfer from kinetic to potential energy. Our current method can not detect this detail. However, given the large uncertainty range associated with the observational $V(r)$, we should not expect to capture this level of detail even if we used an alternate method. The satisfactory fitting using our method implies that obtaining KE flux information from the SF3 is possible.

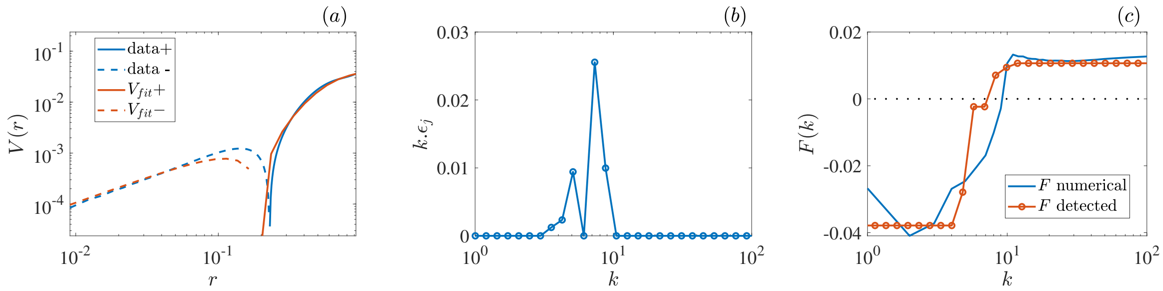

Figure S9: Fitting of the kinetic third-order structure function, the detected energy input and energy flux.
